# Announcement: XXXIII International Conference on Coordination Chemistry

**DOI:** 10.1155/MBD.1997.295

**Published:** 1997


					XXXlll INTERNATIONAL
CONFERENCE ON
COORDINATION
nsCHEMISTRY
"The Chemistry of Metal Io =n Everyday Life"
dedicated to Luigi Sacconi
Centro Congressi e Centro Affari Firenze, Firenze, Italy
August 30 September 4, 1998
CHAIRMAN
Ivano Bertini, University of Florence
Vincenzo Balzani, University of Bologna
Carlo Bellitto, ICMAT CNR, Rome
Claudio Luchinat, University of Florence
SECRETARY
Maurizio Peruzzini, ISSECC CNR, Florence
Carlo Mealli, ISSECC CNR, Florence
Mauro Micheloni, University of Urbino
Andrea Scozzafava, University of Florence
33rd ICCC Secretariat, Department of Chemistry, University of Florence,
Via Gino Capponi, 0121 Firenze, Italy
Telephone +39-55-2757552 (Chairman); +39-55-245990 (Secretary)
Fax +39-55-2478366 or +39-55-2757555
e-mail: iccc@ LRM.FI.CNR.IT; Internet address: http://risc3.1 rm.fi.cnr.it:8002/
Organized by:
Universit& di Firenze, Area della Ricerca di Firenze del CNR, Societ& Chimica Italiana
Sponsored by:
International Union of Pure and Applied Chemistry; Consiglio Nazionale delle Ricerche
Ministero dell'Universit& e della Ricerca Scientifica, Ministero degli Affari Esteri, Comune di Firenze
Important Information and Dates:
All correspondence, regarding submission of abstracts and inquiries MUST BE
ADDRESSED to the 33rd ICCC Secretariat:
33rd ICCC Secretariat, Department of Chemistry, University of Florence,
Via Gino Capponi, 7, I- 50121 Firenze, Italy
The registration form and the payment of the registration fee MUST BE ADDRESSED to
Societ& Chimica Italiana:
331d ICCC Registration Office, Societa Chimica Italiana
Attn. Ms. Valeria Sleiter, Viale Liegi, 48, 00198 Roma, Italy
The Hotel reservation form MUST BE ADDRESSED to the Newtours S. r. I.:
"Newtours/' S.r.l., Att. Ms. Michela Evangelisti, Via San Donato, 22, I- 50137 Florence, Italy
Abstract Submission Deadline
Early Registration Deadline
March 31 st, 1998
March 31 st, 1998
295
For additional information and the latest news, visit the Internet page at the address:
http://risc3.1 rm.fi.cnr.it:8002/
The 33rd ICCC will be held from Sunday, August 30th till Friday, September 4th, 1998. On Sunday,
the activities will take place at the City Hall, Palazzo Vecchio. From Monday through Friday, the
scientific activities will take place at the Centro Congressi e Centro Affari, Piazza Adua 1, close to
the central railway station "Santa Maria Novella".
ABOUT COORDINATION CHEMISTRY
Coordination Chemistry is a branch of Science which regards the species and the reactions
involving metal ions. Historically, it began to develop in the late fifties when the laws of chemical
bonding involving the metal ions begun to be understood. Eventually, this science became the
basis for the investigation and application of industrial catalysis, both in homogeneous and
heterogeneous phases. In fact, most of the homogeneous catalysts are coordination compounds,
typically the titanium-based compounds used in Ziegler-Natta polymerization reactions. In addition,
most of the heterogeneous catalysts involve metal-substrate interactions.
The chemistry of metal ions, which is the essence of this conference as underlined by the subtitle,
has exploded in the last decade due to its implications in new branches of science, including
supramolecular chemistry which is a part of this meeting for what is in common with coordination
chemistry. Typical examples of the chemistry of metal ions are magnetic materials, liquid crystals,
electronic devices (including semi- and super-conductors), materials with non-linear optical
properties, energy storage devices etc. Of course, all of these RTD strategies must take into
account
environmental compatibilities. Therefore great emphasis is given to the interaction of metal ions
with living organisms, and particularly humans, e.g. how do lead and aluminium interact with proteins
and tissues and what is the molecular basis of their toxicity?
Metal ions have quite recently been discovered to have a catalytic role in gene factors, DNA and
RNA replications and in biological catalysis of many reactions in all metabolic processes.
Finally, metal ions have been discovered to play an important part in powerful drugs from the
famous cis-platinum and iron-bleomycin as anticancer drugs, to gold- and copper-containing drugs
such as antiarthrytic. Metal ions are also often used as contrast agents, typically those used in
magnetic resonance imaging.
These are just some glimpses of the impact of coordination chemistry on the many diverse
technological areas.
Plenary Lectures
Claudio Bianchini, ISSECC CNR, Florence, Italy
Kim R. Dunbar, Michigan State University, MI, USA
Odile Eisenstein, University of Montpellier, France
Harry B. Gray, Caltech, Pasadena, CA, USA
Hiizu Iwamura, Kyushu University, Fukuoka, Japan
Stephen J. Lippard, MIT, Cambridge, MA, USA
Robin N. Perutz, University of York, UK
Lawrence Que, Jr., University of Minnesota, MN, USA
Alex von Zelewsky, University of Fribourg, Switzerland
Special Lecture "The View of a Nobel Laureate"
Jean-Marie Lehn, University of Strasbourg, France
Pre-conference Lecture "Metal Ions in Environment and Life"
Helmut Sigel, University of Basel, Switzerland
Special Session "The Way we were"
Fred Basolo, Northwestern University, IL, USA
Stanley Kirschner, Wayne State University, MI, USA
Luigi Maria Venanzi, ETH, Zurich, Switzerland
Shoichiro Yamada, University of Osaka, Japan
296
List of lecturers at the XXXIII ICCC
Tutorials in Advanced Spectroscopy
EPR-ENDOR
Edgar J. J. Groenen, Leiden, The Netherlands
Preedge, EXAFS, XANES
James Penner-Hahn, University of Michigan, USA
A: Supramolecular Chemistry
Nanostructures. Synthesis versus Self-assembly
Peter Belser, Fribourg, Switzerland
Gianfranco Denti, Pisa, Italy
Makoto Fujita, Okazaki, Japan
Jean Pierre Sauvage, Strasbourg, France
Supramolecular Photochemistry
Lucia Flamigni, Bologna, Italy
Masa-aki Haga, Kamihama, Japan
Thomas J. Meyer, Chapel Hill, NC, USA
Daniel G. Nocera, Michigan State, MI, USA
Franco Scandola, Ferrara, Italy
Dendrimers
Sebastiano Campagna, Messina, Italy
Edwin C. Constable, Basel, Switzerland
Jean-Pierre Majoral, Toulouse, France
Nicholas J. Turro, New York, NY, USA
Fritz VSgtle, Bonn, Germany
Supramolecular Switches and Machines
Vincenzo Balzani, Bologna, Italy
A. Prasanna de Silva, Belfast, UK
Luigi Fabbrizzi Pavia, Italy
Supramolecular Electrochemistry
Didier Astruc, Bordeaux, France
Paul D. Beer, Oxford, UK
Michael J. Therien, Philadelphia, PA, USA
Margherita Venturi, Bologna, Italy
Heterogeneous and Sofid State Systems
Donald Fitzmaurice, Dublin, Ireland
Michael Graetzel, Lausanne, Switzerland
Olivier Kahn, Bordeaux, France
Thomas E. Mallouk, Penn State, PA, USA
Molecular Electronics and Non-linear Optics
Luisa De Cola, Bologna, Italy
Jean-Pierre Launay, Toulouse, France
John A. McCleverty, Bristol, UK
Session Lecturers
Hans Gudel, Bern, Switzerland
Fraser J. Stoddart, Birmingham, UK
Jeffrey Zink, Los Angeles, CA, USA
NMR of Paramagnetic Compounds
Claudio Luchinat, Florence, Italy
NMR of Solids
Ann McDermott, Columbia University, NY, USA
B: Coordination Compounds in Material Chemistry"
Molecular Metals and Superconductors
Eric Canadell, Barcelona, Spain
Patrick Cassoux,Toulouse, France
Hayao Kobayashi, Okazaki, Aichi, Japan
Coordination Complexes as Magnetic Materials
George Christou, Bloomington, IN, USA
Peter Day, London, UK
Michael Verdaguer, Paris, France
Masahiro Yamashita, Nagoya, Japan
Intercalation Compounds
Thomas Bein, West Lafayette, IN, USA
Mercouri G. Kanatzidis, Michigan State, MI, USA
Jean Rouxel, Nantes, France
Jean-Marie Tarascon, Amiens, France
Spin Crossover
Marie-Laure Boillot, Orsay, France
Philipp GQtlich, Mainz, Germany
Jaap G. Haasnoot, Leiden, The Netherlands
Andreas Hauser, Geneva, Switzerland
Precursors for electronic Materials
Leonard V. Interrante, Troy, NY, USA
Polynuclear Transition Metal Compounds
James A. Ibers, Northwestern University, IL, USA
Achim MQIler, Bielefeld, Germany
Low-Dimensional Inorganic Solids
Jing Li, Rutgers, NJ, USA
Session Lecturers
Lidia Armelao, Padua, Italy
Renato Ugo, Milan, Italy
Yuri V. Yablakov, Kazan, Russia
297
C: Organometallic Chemistry & Catalysis D: Aqueous Organometallic Chemistry
Computational Methods co-organized by Edward Rosenberg, University of
Thomas Albright, Houston, TX, USA Montana, MT, USA, and Silvio Aime, University of Turin,
Maria Jose Calhorda, Lisbon, Portugal Italy
Peter Comba, Heidelberg, Germany
Alfred Barry P. Lever, Toronto, Canada Mario Bressan, Pescara, Italy
Boy Cornils, Frankfurt, Germany
Metal Complexes of Cyclic Polyenes and Fullerenes Donald J. Darensbourg, College Station, TX, USA
Alan L. Balch, Davis, CA, USA
Jurgen Heck, Hamburg, Germany
Brian F. G. Johnson, Cambridge, UK
John R. Shapley, Urbana, IL, USA
Hubert Wadepohl, Heidelberg, Germany
Main Group Elements
Dainis Dakternieks, Deakin, Australia
Klaus Jurkshat, Dortmund, Germany
Lai Yoong Goh, Kuala Lumpur, Malaysia
Otto J. Scherer, Kaiserslautern, Germany
Kohei Tamao, Kyoto, Japan
Early Transition Metals
John E. Bercaw, Caltech, Pasadena, CA, USA
Richard R. Schrock, MIT, Cambridge, MA, USA
Kazuyuki Tatsumi, Nagoya, Japan
C-C Bond Formation and Bond Breaking
Pierre H. Dixneuf, Rennes, France
Cornelis J. Elsevier, Amsterdam, The Netherlands
John Gladysz, Salt Lake City, UT, USA
David Milstein, Rehovot, Israel
Richard H. Fish, Berkeley, CA, USA
Douglas B. Grotjahn, Tempe, AZ, USA
Philippe Kalck, Toulouse, France
Heinz-Bernhard Kraatz, Ottawa, Canada
Brian F. Hanson, Blacksburg, VA, USA
Bruce M. Novak, Amherst, MA, USA
Charles G. Riordan, Kansas, KS, USA
David R. Tyler, Eugene, OR, USA
Pin Yang, Taiyuan, China
E: Solution Chemistry
Metal Based Drugs (Metals in Medicine)
Sue Berners-Price, Nathan, Australia
Timothy J. Egan, Capetown, South Africa
Bernhardt Lippert, Dortmund, Germany
Kazuko Matsumoto, Tokyo, Japan
Jan Reedijk, Leiden, The Netherlands
Peter J. Sadler, Edinburgh, UK
Roberto Sanchez-Delgado, Caracas, Venezuela
Wenxia Tang, Nanjing, China
Kui Wang, Beijing, China
Lon J. Wilson, Houston, TX, USA
Activation of Small Molecules
Ernesto Carmona, Sevilla, Spain
Jwu-Ting Chen, Taipei, Taiwan
Jose Gimeno, Oviedo, Spain
Iliya I. Moiseev, Moscow, Russia
Transition Metal Hydrides
Kenneth G. Caulton, Bloomington, IN, USA
Guochen Jia, Kowloon, Hong Kong
Contrast Agents in MRI
Silvio Aime, Turin, Italy
Thomas J. Meade, Pasadena, CA, USA
Fulvio Uggeri, Milan, Italy
Solution Supramolecular Chemistry
Cynthia Burrows, Salt Lake City, UT, USA
Daryle H. Busch, Kansas, KS, USA
Thomas A.Kaden, Basel,Switzerland
Dan Meyerstein, Beer-Sheva, Israel
Kenneth N. Raymond, Berkeley, CA, USA
Hydrodesulfurization and Hydrodenitrogenation
Thomas B. Rauchfuss, Urbana, IL, USA
Edward I. Stiefel, Annandale, NJ, USA
Redox Equilibria
Pradyat Banerjee, Calcutta, India
Dieter Sellmann, Erlangen, Germany
Trends and Perspectives in Homogeneous Catalysis Nucleotides
Rumiya R. Abdreimova, Almaty, Kazakhstan Einar Sletten, Bergen, Norway
Peter M. Maitlis, Sheffield, UK Osamu Yamauchi, Nagoya, Japan
Jozef J. Ziolkowski, Wroclaw, Poland
Session Lecturers
Eiichi Kimura, Hiroshima, Japan
Hitoshi Ohtaki, Shiga, Japan
298
F" Bioinorganic Chemistry G" Metals in Life and Environment
Molecular Dynamics Inwlving Metal Ions
Lucia Banci, Florence, Italy
Tim Clark, Erlangen, Germany
Per Siegbahn, Stockholm, Sweden
Metalloproteins
Shigetoshi Aono, Ishikawa, Japan
Gerard W. Canters, Leiden, The Netherlands
Luigi Casella, Pavia, Italy
James A. Cowan, Columbus, OH, USA
Sture Forsen, Lund, Sweden
Anjos L. Macedo, Lisbon, Portugal
Bo G. Malmstrom, Gothenburg, Sweden
Alexander D. Ryabov, Moscow, Russia
Shinnichiro Suzuki, Osaka, Japan
Alejandro J. Vila, Rosario, Argentina
Jay R. Winkler, Caltech, Pasadena, CA, USA
Modeling
Dimitri Coucouvannis, Ann Arbor, MI, USA
Lian-Nian Ji, Guangzhou, China
Kenneth D. Karlin, Baltimore, MD, USA
Lechoslaw Latos-Grazynski, Wroclaw, Poland
Bernard Meunier, Toulouse, France
Vincent L. Pecoraro, Ann Arbor, MI, USA
Giovanni Purrello, Catania, Italy
Heinrich Vahrenkamp, Freiburg, Germany
Ann Walker, Tucson, AZ, USA
Karl Wieghardt, M0hleim, Germany
Session Lecturers
H. O. Allen Hill, Oxford, UK
Edward I. Solomon, Stanford, CA, USA
The Nitrogen Cycle
Barbara K. Burgess, Irvine, CA, USA
Stefano Ciurli, Bologna, Italy
The Nitrogen Activation
Michael D. Fryzuk, Vancouver, Canada
Masanobu Hidai, Tokyo, Japan
Barry E. Smith, Norwich, UK
The Sulfur Geobiochemical Cycle
Antonio V. Xavier, Lisbon, Portugal
Activation of Small Molecules by Iron Centers
Isabelle Artaud, Paris, France
Richard Cammack, London, UK
Isao Morishima, Kyoto, Japan
Hans Toftlund, Odense, Denmark
Speciation
Peter May, Murdoch, Australia
Rudi van Eldik, Erlangen, Germany
David R. Williams, Cardiff, UK
Carbon Dioxide Activation and Functionalization
Michele Aresta, Bari, Italy
Marcetta Y. Darensbourg, College Station, TX, USA
Etsuko Fujita, Brookhaven Nat. Lab., NY, USA
Dorothy H. Gibson, Luisville, KY, USA
Joelle Mascetti, Bordeaux, France
Session Lecturers
William DeW. Horrocks, Jr., PennStateUriversity, PA, USA
Geoffrey A. Sykes, Newcastle, UK
299

				

